# Dendritic Cells and T Cells, Partners in Atherogenesis and the Translating Road Ahead

**DOI:** 10.3389/fimmu.2020.01456

**Published:** 2020-07-29

**Authors:** Li Sun, Wenjie Zhang, Yanfang Zhao, Fengge Wang, Shan Liu, Lei Liu, Lin Zhao, Wei Lu, Minghui Li, Yuekang Xu

**Affiliations:** Anhui Provincial Key Laboratory for Conservation and Exploitation of Biological Resources, College of Life Science, Anhui Normal University, Wuhu, China

**Keywords:** atherosclerosis, dendritic cells, T cells, inflammation, immunotherapy

## Abstract

Atherosclerosis is a chronic process associated with arterial inflammation, the accumulation of lipids, plaque formation in vessel walls, and thrombosis with late mortal complications such as myocardial infarction and ischemic stroke. Immune and inflammatory responses have significant effects on every phase of atherosclerosis. Increasing evidence has shown that both innate and adaptive “arms” of the immune system play important roles in regulating the progression of atherosclerosis. Accumulating evidence suggests that a unique type of innate immune cell, termed dendritic cells (DCs), play an important role as central instigators, whereas adaptive immune cells, called T lymphocytes, are crucial as active executors of the DC immunity in atherogenesis. These two important immune cell types work in pairs to establish pro-atherogenic or atheroprotective immune responses in vascular tissues. Therefore, understanding the role of DCs and T cells in atherosclerosis is extremely important. Here, in this review, we will present a complete overview, based on existing knowledge of these two cell types in the atherosclerotic microenvironment, and discuss some of the novel means of targeting DCs and T cells as therapeutic tactics for the treatment of atherosclerosis.

## Introduction

The term “atherosclerosis” is derived from the Greek word “atheroma” meaning “soft or porridge-like” to describe the physical appearance of the intima of arteries, which was first named by Felix Marchand in 1904 to account for almost all obstructive processes in the arteries (1). Being one of the leading causes of morbidity and death from cardiovascular diseases (2), atherosclerosis involves a chronic inflammatory process within the large and medium-sized arterial walls that leads to characteristic plaque formation, rupture, and ischemic injury of the dependent vascular bed (3, 4). As a result, this tissue damage will further trigger immune responses. Both innate and adaptive immunity have played important roles in the initiation and development of atherosclerosis (5–7). The initial step in the vascular pathology involves their increased permeability for plasma lipid components, like low-density lipoprotein (LDL) the abbreviated form can be looked up in Table 1, in the presence of cardiovascular risk factors, such as hyperlipidemia, hypertension, diabetes, smoking, and obesity, etc. (4). This abnormal deposition of lipids on the arterial wall initiates the recruitment of leukocytes and accumulation of oxidized LDL (oxLDL) within the intima of arteries. Loaded with oxLDL and other lipids, arterial monocytes, accumulate within nascent lesions of vascular walls and transform into foam cells to constitute early atherosclerotic plaque (8).

**Table 1:** Abbreviated form.

Antigen-presenting cells	APC
Arterial tertiary lymphoid organ	ATLO
Basic leucine zipper ATF-like transcription factor 3	BATF3
Blood dendritic cell antigen	BDCA
Bone marrow derived DCs cultured with GM-CSF	GM-DCs
C-C motif ligand	CCL
Coronary artery disease	CAD
Cytometry by time of flight	CyTOF
Cytotoxic T-lymphocyte-associated protein 4	CTLA4
Dendritic cells	DCs
FMS like tyrosine kinase 3 ligand	Flt3L
Forkhead/winged helix transcription factor 3	Foxp3
Helper T cell	Th
High-sensitivity C-reactive protein	hsCRP
Inhibitor of DNA binding 2	ID2
Indoleamine 2,3-dioxygenase	IDO
Interferon	IFN
Immunoglobulin M	IgM
Interleukin	IL
Interferon regulatory factor	IRF
Lineage	Lin
Low-density lipoprotein	LDL
Low-density lipoprotein receptor	Ldlr
Macrophage colony stimulating factor	M-CSF
Malondialdehyde-modified LDL	MDA-LDL
Myeloid DCs	mDCs
Oxidized low-density lipoprotein	oxLDL
Plasmacytoid DCs	pDCs
Plasmacytoid DC antigen-1/marrow stromal cell antigen 2	PDCA1/BST2
Programmed cell death protein 1	PD-1
Regulatory T cells	Tregs
Reticuloendotheliosis viral oncogene homolog B	RELB
Retinoic acid receptor-related orphan receptors	ROR
Secondary lymphoid organ	SLO
Smooth muscle cell	SMC
Signal transducer and activator of transcription	STAT
Single-cell RNA-sequencing	scRNAseq
Single-nucleotide polymorphisms	SNPs
T-box transcription factor-21	T-bet
T-cell factor 4/E2-related factor 2	TCF4/ E2-2
Transforming growth factor	TGF
Toll-like receptor	TLR
Tumor necrosis factor	TNF
Vascular-associated lymphoid tissue	VALT
Vascular cell adhesion molecule 1	VCAM-1

*This table identifies the full name of the abbreviation in the article*.

Within the mononuclear phagocytic myeloid cell lineages, accumulating evidence suggests that DCs, a unique type of innate immune cells, play an important role as central instigators of the immune response within the arteries, and are involved in both promoting and inhibiting atherogenesis (9). Partly as a result of the accumulation of inflammatory monocytes and activated DCs, adaptive immune cells such as helper T cells (Th1, Th2, Th17) and regulatory T cells (Tregs) can also be detected within atherosclerotic lesions with each different class of T cells having different effect on atherogenesis (10). Despite their accumulation in the diseased locus, how DCs influence the initiation and progression of adaptive immune responses in atherosclerosis remains elusive; nor has the cause of the T cell activation in plaques been clarified in detail.

In this review, we will review selective parts of innate and adaptive immune processes in the pathogenesis of atherosclerosis, as well as their connections that have recently come to light as affecting atherogenesis, mainly focusing on the role of different subsets of DCs and T cells, and discuss different schools of thought or controversies. Finally, novel means of targeting DCs and T cells as therapeutic tactics with any research gaps will also be summarized for potential development in the treatment of atherosclerosis.

## Basic Immunological Features of Vascular Tissues

With the development of modern sciences, more and more studies show that vascular tissues might possess immunological potentials, although they are traditionally included in the circulatory system. Anatomically, large vascular walls are surrounded and nourished by numerous capillaries and lymphatic vessels containing T cells, mast cells, macrophages, and immature DCs (11). As a consequence, the infiltration of immune cells in vascular tissues occurs as early as the first year of childhood in humans (12). However, different from secondary lymphoid tissues, the vascular tissues contain more innate immune cells than adaptive immune cells, which can form their own unique immune network.

The immunological potential of the vascular tissue represents far more than merely accumulation of immune cells. What is more, the immune cells within the distinct microenvironment of vascular tissues can form immunologically functional units. Subjected to multiple stimulation by vascular risk factors, oxLDL can trigger the migration of DCs from adventitia to intima and settle down in areas vulnerable to the impact of blood flow, where they recruit mononuclear immune cells and gradually form structure named vascular-associated lymphoid tissue (VALT) or arterial lymphoid organ (ATLO) adjacent to the diseased locus (13). Many pro-inflammatory and anti-inflammatory immune cells stay in ATLO as they are in secondary lymphoid organs (SLO) such as the spleen and lymph nodes. However, the types and proportions of cells are remarkably different. For example, DCs in ATLO are mainly myeloid DCs derived from mononuclear cells and the proportion of macrophages is low, whereas in SLO, the number of B cells and macrophages are abundant but DCs are scarce (14), which suggests that vascular DCs play an important role in local immunity. Consistently, DCs were found to be significantly more frequent in atherosclerotic plaques nearby ATLO than other part of the vascular tissues in human samples (15).

## Roles of Different Subsets of Vascular Dendritic Cells in Atherosclerosis

DCs are professional antigen-presenting cells (APCs) that facilitate the development and progression of atherosclerosis. They are identified within healthy aorta, but increase in number during disease progression (16, 17). So far, several subsets of DCs have been identified in the artery tissues of C57BL/6 mice by phenotypical and functional testing. An important question derived is whether these different vascular DC subsets play different functions in atherogenesis. The following sections will summarize the current knowledge of their phenotype and origin and further discuss the functional roles of these cells, although definitive effects of each subset in disease progression remains to be elucidated and some of them are controversial (Table 2).

**Table 2:** Distinct vascular DC subsets and their T cell partners in atherogenesis.

	**CD103^**+**^DCs**	**CD11b^**+**^DCs**	**CCL17^**+**^DCs**	**pDCs**
**Number**	Scarce	Abundant	Scarce	Less than CD103^+^DCs
**Location**				
Healthy	Intima, Aorta sinus	Intima	None	Intima
AS	Overall aorta	Overall aorta	Atherosclerotic lesions in the aortic root	Shoulder regions of atherosclerotic lesions
**Transcription factors required for development**	BATF3, IRF8, ID2	RELB, RBPJ, IRF4, Notch2	?	TCF4/E2-2, IRF8
**Cell surface markers**	CD11c, MHCII, Clec9A, DEC205, CD103	CD11c, MHCII, CD11b F4/80, CX3CR1, DC-SIGN	CD11c, MHCII, CD205 CD11b, CCR7	CD11c^int^, CD45RA PDCA-1, SiglecH
**Dependent cytokines**	Flt-3L	M-CSF?	?	Flt-3L
**Ontogeny**	Flt-3L-dependent pre-DC precursors	Monocytes/pre-DCs	?	Common DC precursors
**Signature cytokines**	?	?	CCL17	IFN-α/β
**Downstream T cell partners**	Tregs	Teff/Tregs	Teff /Tregs	Teff
**Animal model and its effects on AS**	Flt3^−/−^Ldlr^−/−^mice (18) CD103^+^DCs↓ Treg/IL-10↓ 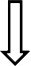 Aggravated AS IRF8^fl/fl^CD11c^Cre+^Ldlr^−/−^ mice (19) CD103^+^DCs↓ T & B cell activation↓ 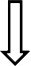 Reduced AS Batf3^−/−^ApoE^−/−^mice (20) CD103^+^DCs↓ Th1/CCL5↓ 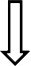 Reduced AS	ApoE^−/−^ mice (21–23) CD11b^+^DCs↑ exosome↑ T-bet &CCR5&CCR7↑ 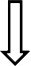 Aggravated AS CD11b-DTR ApoE^−/−^ Mice (24) CD11b^+^DCs↓ Mφ number & function 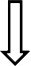 Reduced AS	CCL17^−/−^ApoE^−/−^ mice (25) CCL17^+^DCs↓ Mφ & T cell↓ 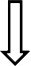 Reduced AS CCL17 neutralization in ApoE^−/−^ mice (26) CCL17↓ Treg/IL-10↓ 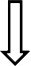 Reduced AS	CD11c^Cre^TCF4^−/fl^Ldlr^−/−^ mice (27) pDCs↓ MHCII, IFN-γ &T cell↓ 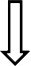 Reduced AS pDCs depletion in ApoE^−/−^ mice (28) pDCs↓ T cell&Mφ↓ 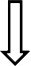 Reduced AS pDC delpletion in Ldlr^−/−^ mice (29) pDCs↓ T cell proliferation, IFN-γ↑ 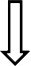 Aggravated AS
**Possible pathways**	TGF-β/retinoic acid and CCL22	CCR5&CCR7?	CCR4 and IL-2/STAT5/Tregs	IDO/Tregs
**Roles in AS**	Anti-atherogenic Pro-atherogenic	Pro-atherogenic	Pro-atherogenic	Anti-atherogenic Pro-atherogenic

*AS, atherosclerosis; BATF3, basic leucine zipper ATF-like transcription factor 3; IRF, interferon regulatory factor; IFN, interferon; ID2, inhibitor of DNA binding 2; RELB, reticuloendotheliosis viral oncogene homolog B; TCF4/E2-2, T-cell factor 4/E2-related factor 2; Flt-3L, FMS like tyrosine kinase 3 ligand; M-CSF, macrophage colony stimulating factor; Teff, effector T cells; Mφ, macrophage; IL, interleukin; TGF, transforming growth factor; CCL, C-C motif ligand; ApoE, Apolipoprotein E; Ldlr, low-density lipoprotein receptor; IDO, indoleamine 2,3-dioxygenase; STAT5, signal transducer and activator of transcription 5*.

### CD103^+^DCs in Atherosclerosis

Located in the intima of healthy aorta, CD103^+^DCs were believed to be a subset with immune regulatory functions, although these cells increased in the overall aorta during atherosclerosis (18). Vascular CD103^+^DCs are like their other tissue counterparts in phenotypes. For example, they express cell surface markers CD11c and MHCII, but lack the expression of macrophage makers like CD11b, CX3CR1, and F4/80 (18). CD103^+^DCs need transcription factors such as basic leucine zipper transcription factor, ATF-like 3 (BATF3) (30), interferon regulatory factor 8 (IRF8) (31), and inhibitor of DNA binding 2 (Id2) (32) for their development. In addition, the CD103^+^DCs have similar gene signatures to that of CD8^+^DCs in the spleen, and both are FMS like tyrosine kinase 3 ligand (Flt-3L)-dependent (33). In line with these findings, the CD103^+^DCs subset in the aortic wall expanded in response to Flt-3L injection, and was absent in mice deficient in Flt-3, indicating that the development of the CD103^+^DCs requires Flt-3/Flt-3L interaction, and they are derived from classical Flt3L-dependent pre-DC precursors (18). Recently, a lineage tracing study revealed that the CD103^+^DCs in the cardiovascular tissues could be developed from an intermediate CD103^−^CD11b^−^DC subset that expressed IRF8 and expanded readily in response to Flt-3L administration (34).

With a typical “dendritic” morphology as in classical DCs, intimal CD103^+^DCs exhibit classical DC functions in activating allogeneic T cells (18). It has been reported that in Flt3^−/−^Ldlr^−/−^ mice, depleted CD103^+^DCs due to Flt3 deficiency aggravated plaque burden, accompanied by decreased aortic Tregs, limited interleukin-10 (IL-10) secretion, and increased production of interferon-γ (IFN-γ) and tumor necrosis factor-α (TNF-α) without significant alterations in the lipid levels (18), indicating an immune suppressive role for this DC subset in atherosclerosis. Interestingly, CD103^+^DCs in the gut were found to be potent inducers of Treg, partly via secretion of transforming growth factor-β (TGF-β) and retinoic acid (35). In addition, lung CD103^+^DCs also demonstrated their unique ability to recruit circulating peripheral Tregs through chemokine CCL22 (36). Collectively, these data of CD103^+^DC in other tissues lead to a hypothesis that vascular CD103^+^DCs could also induce Treg differentiation and recruitment in early atherosclerotic lesions via TGF-β/retinoic acid or CCL22 pathways, respectively, which agrees with previous reports that vascular CD103^+^DCs protect against atherosclerosis via Tregs (18). However, when the atherogenic roles of CD103^+^DCs were assessed in mice deficient in their dependent transcription factor Batf3 in ApoE^−/−^ mice, which also lacked CD103^+^DCs, a significant reduction in atherogenesis via reduced Th1 cell induction and C-C motif ligand 5 (CCL5) expression were found (20), whereas mice deficient in the same transcription factor but crossed with Ldlr^−/−^ mice demonstrated mild effects on the immune response in the spleen without altering atherosclerotic lesion formation and plaque phenotype (37). These conflicting results could be caused by different model specific pathologies in the development of atherosclerosis. In addition, since Flt3 signaling alters many other immune cell populations and biological processes, whole body deficiency in these growth factors or transcription factors may have affected other cells as well (38). For example, during the inflammation with elevated IL-12 and IFN-γ production, other DC subsets also developed in Batf3^−/−^ mice (39). Therefore, a lineage specific depletion model is necessary to accurately identify the role of CD103^+^DC subset in atherosclerosis.

It has been demonstrated that restricted deletion in hemopoietic cells of IRF8, a specific transcription factor for the development of lymphoid CD8^+^cDCs and aortic CD103^+^DCs, led to reduced atherosclerosis via a severe suppression of T and B cell activities, although plasma cholesterol levels were increased (19), suggesting that lymphoid CD8^+^cDCs and aortic CD103^+^DCs can promote the development of atherosclerosis via activating adaptive components. These studies established a critical role in proatherogenic immunity of this DC subset, which could be targeted to modulate the development of vascular diseases.

Apart from pro-inflammatory responses, necrotic core formation as a result of immune destructions promotes plaque development and instability in atherosclerosis. Recently, defective acquisition and presentation of dead cell-associated antigens, a process called efferocytosis, was found in atherosclerotic lesions (40). Interestingly, DCs were professional phagocytes in efferocytosis (41, 42), in which CD103^+^/ CD8^+^DC subsets were proven to be the best (43), as these DC lineages preferentially express C-type lectin receptor Clec9A (also known as DNGR-1), that recognize a preformed signal exposed on dead cells and is required for cross-presentation of dead cell- associated antigen *in vitro* and immunogenicity *in vivo* (44). Consistently, recent studies have demonstrated that specific deletion of Clec9A significantly increases IL-10 expression, reduces macrophage and T-cell contents within the lesions, and limits the development of atherosclerosis (45), re-emphasizing the promoting roles of CD103^+^DC subset in the development of atherosclerosis.

### CD11b^+^DCs in Atherosclerosis

CD11b^+^DCs belong to the most abundant DC subset found in mouse aorta and which reside primarily in intima (18). As one of the DC subsets that emigrate from atherosclerotic plaques under normolipidemic conditions, CD11b^+^DCs are drained to local lymph nodes via the afferent lymphatics (46). The differentiation of CD11b^+^ conventional DCs (cDCs) is controlled by transcription factors such as reticuloendotheliosis viral oncogene homolog B (RELB) (47), NOTCH2 (48), RBPJ (49), IRF2 (50), and IRF4 (51). Of note, IRF4 also controls functional aspects of CD11b^+^cDCs, such as their MHC presentation (52) and migration (53). In addition, monocyte-derived CD11b^+^DCs (CD11b^+^ mDCs) were also identified in atherosclerotic vessel walls by their dependence on macrophage colony-stimulating factor (M-CSF) 1 receptor (18) or expression of CD64 (21). Like their counterparts in other tissues, the vascular CD11b^+^DCs express many other common macrophage markers such as F4/80, CD115, CX3CR1, and C-type lectin DC-SIGN (54). CD11b^+^cDCs can be characterized by their production of cytokines, such as IL-6 (55) and IL-23 (56), whereas CD11b^+^mDCs are unique to TNF-a and IL-10 secretion (57). A profound reduction but not complete ablation in total resident vascular CD11b^+^DCs were found in mice deficient in monocytes and macrophage-dependent cytokine M-CSF (18), confirming the heterogenous origins of this DC subset. Consistently, adoptive transfer studies have demonstrated that tissue CD11b^+^DCs populations can be derived *in vivo* from pre-DCs and monocytes in the liver, lung, and kidney (58). Despite their developmental potential from pre-DCs in the absence of M-CSF, Flt3 deficiency in mice did not affect CD11b^+^cDCs numbers in vascular tissue, indicating that this vascular CD11b^+^cDCs might have a different cytokine signaling requirement to that of vascular CD103^+^DCs, driving different sets of transcription factors along their developmental pathways (18).

Unlike that of CD103^+^DCs, the functions of vascular CD11b^+^DCs were found to be associated with the local T cell expansion (59), indicating that CD11b^+^DCs may regulate T cell homeostasis in vascular tissues. In terms of disease correlation, CD11b^+^DCs have been shown to rapidly increase in mouse atherosclerotic plaques during atherogenesis (21). Parallel to mice, CD11b^+^DCs were also observed to be increased in human plaques. Remarkably, plaques from distinct anatomical locations exhibited different cellular compositions: carotid plaques contained more CD11b^+^DCs than femoral plaques (60). Functionally, isolated aortic CD11b^+^DCs have been shown to exhibit cardinal DC functions, such as the capacity to activate whole allogeneic CD4^+^T cells *in vitro* as efficiently as splenic cDCs (18). Moreover, conditional deletion of CD11b^+^ monocytes, the precursors of CD11b^+^mDCs, mitigated plaque development and altered plaque composition in atherosclerotic mice, suggesting a possible involvement of CD11b^+^mDCs in the disease (24). At molecular and cellular levels, CD11b^+^DCs were found to promote the development of atherosclerosis by either exosome membrane-bound TNF-α that triggers inflammation in recipient endothelial cells (22), or interacting with circulating natural killer T cells (NKT) and Tregs (23). Collectively, these data indicate that CD11b^+^DCs aggravated the inflammatory status in atherosclerotic plaque.

Autophagy has recently emerged as a major modulator of a variety of cellular functions with high relevance to the development and progression of atherosclerosis (61). A recent study revealed that CD11b^+^DC deficient in an autophagy-related molecule Atg16L that binds ATG5 and links the isolation membrane to the formation of the autophagosome (62), developed a TGF-b-dependent tolerogenic phenotype and promoted the expansion of Tregs, whereas no such effects were seen with *Atg16l1* deficient CD8a^+^DCs (63), suggesting an essential role of Atg16L complex in the suppressive function of CD11b^+^DCs via autophagy. Consistently, the expanded aortic Tregs *in vivo*, limited accumulation of Th1, and reduced development of atherosclerosis in *CD11c*^*Cre*−^*Atg16l1*^*flox*/*flox*^*Ldlr*^−/−^ mice (*Atg16l1* deletion in total DCs) were proven to be caused by CD11b^+^DC subset, as no such effects were seen when *Atg16l1* was deleted selectively in conventional CD8a^+^DCs and CD103^+^DCs (63). Collectively, these results indicated an essential role of CD11b^+^DC subset in an autophagy-mediated, Treg dependent, pro-atherosclerotic effect.

### CCL17^+^DCs in Atherosclerosis

A recent study reported the presence of another specific subset of DCs, CCL17^+^DCs, in atherosclerotic lesions at the aortic root but not in the healthy vascular wall (25). These lesion resident CCL17-secreting DCs bear a CD11c^+^MHCII^+^CD11b^+^ phenotype but do not express CD115 or F4/80, differentiating them from other CD11b^+^DCs. Like other mature DC subsets, CCL17^+^DCs display an increased expression of costimulatory makers CD40, CD80, and CD86 (64), indicating their capacity to activate CD4^+^T cells *in situ*. Indeed, multiphoton microscopy studies showed that CCL17^+^DCs colocalize with CD4^+^T cells in atherosclerotic lesions and form distinct immune synapses with them (25). However, direct examination of the CD4^+^T- cell priming capacity of CCL17^+^DCs showed that ovalbumin-loaded CCL17^+^DCs exhibited a reduced capacity to activate ovalbumin-specific OT-II T-cells compared with splenic cDCs (25). Notably, these CCL17^+^DCs also express high levels of CCR7, a chemokine receptor mostly expressed in DCs for their ability to migrate (25).

In spite of its limited capacity to directly activate antigen specific T cells, CCL17^+^DCs were found to have a pro-atherogenic role in limiting Tregs expansion and increasing plaque burden, because CCL17 deficient atherosclerotic mice displayed reduced T cell contents due to Treg expansion in the atherosclerosis plaque (25). Since it has been demonstrated that the enhanced T cell number and activities within atherosclerotic lesions were associated with an enhanced atherosclerotic plaque growth (65, 66), these data suggested that CCL17^+^DCs promote atherosclerosis. In line with this notion, administration of a blocking antibody to neutralize CCL17 could protect ApoE^−/−^ mice from lesion formation (26). Consistent with murine samples, increased myeloid DCs and CCL17 expression were also observed in progressive human plaques (67–69). Furthermore, enhanced CCL17 mRNA in human carotid endarterectomy specimens over healthy controls (25) underscores the possible relationship between CCL17^+^DCs and the disease severity.

At molecular levels, CCL17 was previously shown to activate the chemokine receptor CCR4 on T cells as its ligand and to attract not only Th1 and Th2 cells, but also Tregs (70–72). The underlying mechanisms for the CCL17-expressing DCs to mediate these effects on T cells, however, remain unclear. Although a role of CCR4 in recruiting both immune stimulatory and regulatory CD4^+^T cells has been demonstrated *in vitro* (70), the predominant role of this receptor could not be confirmed *in vivo*. Moreover, the phenotypes of CCR4 knock-out mice did not resemble that of CCL17 deficient mice, and lesion formation was similar between chimeric Ldlr^−/−^ mice reconstituted with CCR4^+/+^ and CCR4^−/−^ bone marrow (25). Thus, CCL17 does not seem to work solely through CCR4, suggesting a possible involvement of other CCL17 receptors in the complex functional profile of CCL17. In any cases, downstream of its receptor including CCR4 and/or other surface molecules on T cells, CCL17 may interfere with signal transducers and activators of transcription 5 (STAT5) phosphorylation. Given the essential roles of IL-2 in STAT5 activation for Foxp3 expression in Tregs (73), interference with STAT5 in the CCL17 pathway may affect both T cell proliferation and Treg conversion.

### Plasmacytoid DCs in Atherosclerosis

Plasmacytoid DCs (pDCs) are present in the shoulder regions of both human and mouse atherosclerotic lesions, albeit in smaller numbers than CD103^+^DCs (74, 75). Unlike CD103^+^DCs, pDCs express lower levels of CD11c and MHCII, but are positive for PDCA-1 and SiglecH (76). pDCs are derived directly from the common DC precursors in the bone marrow via a Flt3L-dependent manner, and they migrate to lymphoid and non-lymphoid tissues, including vascular walls, via the blood as pDCs, rather than as precursor cells to be differentiated further in the local tissues (77). Different from cDCs that require specific transcription factor Zbtb46 for development, pDCs are dependent on the helix-loop-helix transcription factor E2-2 (also known as TCF4) (78), which facilitates pDC development by directly suppressing the expression of Id2 and IRF8 (79) (transcription factors critical for CD103^+^DC development). The subset of pDCs are best characterized by their unique functional property to rapidly produce large amounts of type I IFNs and other cytokines during infection (80), a direct consequence of their constitutive IRF7 expression (81). Interestingly, the numbers of circulating pDCs in the blood was negatively correlated with the cardiovascular events in coronary artery disease (82).

The essential roles of pDCs in atherosclerosis have recently been identified by several studies. For example, selective genetic deletion of TCF4 in CD11c^+^ cells significantly reduced Th1 responses and limited atherosclerosis in CD11c^Cre^ TCF4^−−/flox^ Ldlr^−/−^ mice (27). Furthermore, pDC-specific deletion of MHCII molecules also reduced the development of atherosclerosis via defective activation of antigen specific CD4^+^T cells, accompanied by a marked reduction of the T cell-produced inflammatory cytokine IFN-γ and T cell migration into the lesion (27). In response to type A oligo dinucleotides (CpGs), plaque-residing pDCs accelerated the production of TLR4, TNF-α, and IL-12 from myeloid DCs and enhanced CD8^+^ T cell functions within human plaques (75, 83). Collectively, these data suggest pro-atherogenic roles of this DC subset, at least partly via MHC-Ag presentation. In line with the pro-inflammatory effects of pDCs, specific stimulation with CpG or IFN-α enhanced atherosclerotic lesion formation in ApoE^−/−^ mice (74), similar to the observation from another group with the other type I interferon, IFN-β, which accelerated atherosclerotic lesion formation (84).

Unfortunately, multiple studies via antibodies targeting plasmacytoid DC antigen-1/marrow stromal cell antigen 2 (PDCA1/BST2) for pDC depletion produced conflicting results. A reduced lesion formation was found in the ApoE^−/−^ mice with the pDC depletion, which was associated with compromised T cell activity, attenuated levels of pro-inflammatory cytokines, and generally diminished contents of macrophages (28). However, in another study, pDCs depletion in Ldlr^−/−^ mice aggravated atherosclerosis with increased T cell proliferation and elevated IFN-γ production (29), suggesting a regulatory role for pDCs in atherosclerosis. These seemingly opposite outcomes in lesion formation could be associated with different depleting antibodies, the dose, the interval of administration, or even different mouse strain used in these studies, demonstrating a complex and case-dependent role of this DC subset in atherosclerosis. Among these controversies, later works, however, seemed to be in favor of an atheroprotective role of pDC with a mechanism via indoleamine 2,3-dioxygenase (IDO), as pDC was demonstrated to reduce atherosclerosis by suppressing splenic CD4^+^T cell proliferation and activity in an IDO-dependent manner (29), and Ido1^−/−^ atherosclerotic aorta possessed 40% fewer Treg numbers than that of WT controls, suggesting essential interaction of IDO-expressing pDCs with Tregs for the homeostasis of these cell populations in diseased aorta (85). However, a direct link between pDC-restricted expression of IDO and an atheroprotective role has not been established yet. Overall, these data revealed that the role of pDCs in mediating vascular inflammation are intricate and context dependent. Therefore, further comprehensive studies are needed to understand the exact functions of pDCs in atherosclerosis under specific pathological conditions.

## Roles of Different T Cell Subsets in Atherosclerosis

**Figure 1:**
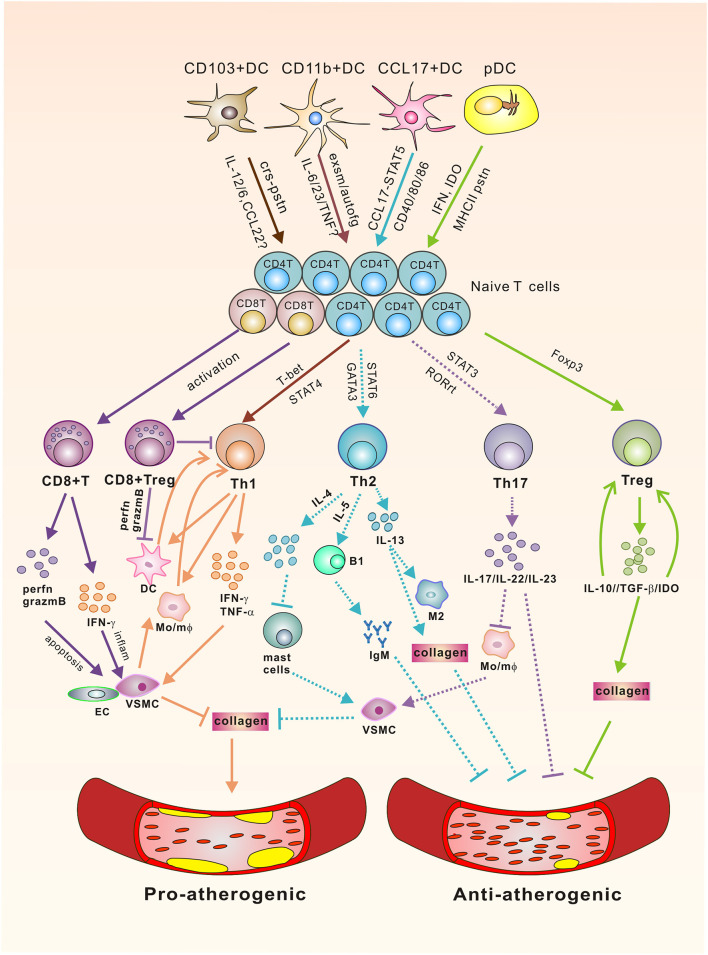
The Link of DCs/T cells and Atherosclerosis. Vascular DC subsets interact with their downstream T cells via polarizing cytokines, co-stimulatory molecules, chemokines, dioxygenases, exosomes, or MHC-II to affect the activation and differentiation of T effector cells via signaling through corresponding transcription factors for various atheroclerotic outcomes. Pro-atherogenic: CD8^+^ T cells promote atherosclerosis via perforin/granzyme B-mediated aposptosis of VSMC/EC and IFN-γ mediated local inflammation. Th1 cells secrete pro-inflammatory cytokines to promote plaque rupture through their effects on VSMCs, which inhibit the synthesis of collagen to destabilize the thick fibrous cap of the plaque, and activate DC/mφ for sustaining the pathogenic Th1 responses. Anti-atherogenic: CD8^+^Tregs can direct their cytotoxic activity toward DCs and also inhibit the differentiation of CD4^+^T cells toward pathogenic subsets. CD4^+^Tregs produce IL-10, TGF-β, and IDO. Of which IL-10 promotes Treg differentiation, whereas TGF-β enhances the survival and proliferation of collagen, rendering a more stable plaque phenotype. IDO shifts the Treg/Teff cell proportion toward a Treg phenotype. Controversial: Experiments with IL-17A knockout mice or inhibition of IL-17A have yielded inconsistent results. Th2 cells produce signature cytokines IL-4 and IL-5, and IL-13. Of these, IL-5 promotes the development of IgM-secreting B1 cells for the clearance of foam cells. Lack of IL-4, however, decreases lesion formation, followed by the activation of mast cells that promote the apoptosis of VSMC, and increases the production of proteases, thereby potentially destabilizing the plaque. IL-13 was identified to have an atheroprotective role through regulating macrophage activities for oxLDL clearance and increasing collagen contents in plaque composition. Solid lines stand for either pro-atherogenic or anti-atherogenic roles, whereas dotted lines represent controversial roles in atherogenesis. crs-pstn, cross-presentation; exsm, exosome; autofg, autophagy; perfn, perferon; grazmB, grazyme B; VSMC, vascular smooth muscle cell; EC, endothelial cells; IDO, indoleamine 2,3-dioxygenase, Mo/mφ, monocyte/macrophage.

The immune responses in atherosclerosis are complete processes. Apart from innate phagocytosis of lipids (86) and, more recently, efficient clearance of microorganisms from the intima (87), DCs are mostly involved in adaptive immunity in the local vascular tissues for inflammation induction, because DCs are the only antigen presenting cells that can activate naïve T cells, which, after differentiation, will be the effector cells of multiple DC subsets in atherosclerosis. Along this line, T cells have been identified in atherosclerotic plaques together with the DCs and play a pivotal role in the progression of atherosclerosis. In fact, accumulating data from laboratories clearly indicated that specific subsets of T cells, as partner cells of DCs, also exert either protective or promoting effects on atherogenesis. The following sections will discuss the current knowledge of different T cell subsets downstream of DCs in atherosclerosis, which is summarized in Figure 1.

### CD4^+^T Cells in Atherosclerosis

While human atherosclerotic lesions have equal numbers of CD4^+^ and CD8^+^T cells, the diseased samples from their mouse counterparts were penetrated predominately by the former (88). Naïve CD4^+^T cells differentiate into various effector subsets, such as Th1, Th2, Th17, and Tregs, depending on the specific microenvironment of different cytokines, signaling through corresponding transcriptional factors.

#### Th1 Cells

Th1 commitment occurs in the presence of IL-12 and IL-18, when these cells start to express the defining transcription factors: signal transducer and activator of transcription 4 (STAT4), T-box transcription factor-21 (T-bet), and secrete the signature cytokines IFN-γ, TNF-α, and IL-2, but downregulate IL-4 and IL-5 (89).

Th1 cells are the most abundant T cell subset that drives the inflammatory responses in atherosclerosis (90). It has been demonstrated that IFN-γ, the hallmark cytokine produced by Th1 cells, has a pro-atherosclerotic role in affecting several cells such as vascular smooth muscle cells (VSMC), monocytes/macrophages, and DCs in atherosclerotic lesions (7). For example, IFN-γ recruits VSMCs to impede collagen synthesis, which will destabilize the thick fibrous cap of the plaque and may lead to rupture. Furthermore, the enhanced activities of monocytes/macrophages and DCs by IFN-γ supports the persistence of the pathogenic Th1 response (91).

Exogenously administered recombinant IFN-γ have been demonstrated to result in a 15% increase in atherosclerotic lesion size (92). The pro-atherogenic role of this Th1 cytokine was also shown by loss-of-function experiments. The absence of IFN-γ or its receptor in ApoE^−/−^ mice (93) mitigated lesion formation and reinforced plaque stability with accelerated collagen synthesis. Likewise, deficiency of T-bet in Ldlr^−/−^ mice (94) also resulted in a 30% reduction of atherosclerosis, accompanied by a concurrent increase of Th2 cytokine (IL-4, IL-5, and IL-10) and immunoglobulin M (IgM) (an atheroprotective natural antibody up-regulated with IL-5) (95). IFN-γ mediates its pro-inflammatory effects mostly through activation of transcription factor STAT1 (96). Consistently, STAT1-deficient ApoE^−/−^ chimeric mice demonstrated smaller plaque areas than that of control mice (97, 98).

Apart from the cytokine mediating the functions of Th1 cells, the cytokines essential for its development and differentiation, such as IL-12 and IL-18, also produce pro-atherogenic outcomes for this T-cell subset. Exogenous administration of IL-12 increased atherosclerotic plaques in the aorta (99), which was in accordance with the finding that administration of anti-IL-12 antibodies into atherosclerotic mice led to a decrease in atherogenesis by 68% with an increase in collagen contents (100). In another study, ApoE^−/−^ mice with a genetic deficiency in IL-12 also demonstrated an alleviated lesion formation in the aortic root at early stages (101). With regard to the role of IL-18 in atherogenesis, less disease was found in ApoE^−/−^ mice when a plasmid encoding a soluble, recombinant IL-18-binding protein was administrated to block bioavailable IL-18 (96), whereas direct injection of IL-18 recombinant protein accelerated atherosclerosis (102). Furthermore, the deficiency of IL-18 in ApoE^−/−^ mice led to a reduction in lesion size of 35% although the proportion of SMC was increased. Collectively, these data suggested that the IL-18/IL-12-Th1-IFN-γ axis is pro-inflammatory and contributes to atherosclerotic lesion development.

#### Th2 Cells

Th2 cells develop in response to IL-4, IL-5, and IL-13, and express the defining transcription factor GATA-binding protein 3 (GATA-3), which, through a positive feedback loop, initiates the secretion of signature cytokines IL-4, IL-5, and IL-13 but inhibits IFN-γ expression (103, 104).

The role of Th2 subsets in atherosclerosis varies based on the different stages and sites of the lesion, and sometimes on different experimental models as well. The major cytokines, IL-4, IL-5, and IL-13, produced by Th2 cells are all considered atheroprotective in some studies. For example, genetic deletion of *il4* accelerated atherosclerotic lesions to a small extent in ApoE^−/−^ mice (101). IL-5 was found to facilitate the development of B1 cells that can produce oxLDL-specific IgM to assist in clearing lipids and reducing local foam cell formation (95), whereas IL-13 was identified to have an atheroprotective role through regulating macrophage activities for oxLDL clearance and increasing collagen contents in plaque composition (104).

In contrast to the atheroprotective effects of IL-4 observed in ApoE^−/−^ mice, a lack of this cytokine in Ldlr^−/−^ mice led to a decreased lesion formation (103), followed by the activation of mast cells that secrete soluble factors to promote SMC apoptosis, reduce collagen synthesis, and increase protease secretion, thus potentially destabilizing the plaque (105). Collectively, these loss of function phenotypes suggested a pro-atherogenic role for IL-4. However, exogenous injection or genetic deletion of *il4* in another study did not affect lesion development regardless of the induction of atherosclerosis by a high fat diet or angiotensin II, indicating a possible case-specific requirement of the Th2 cytokine in atherosclerosis progression (106).

Since different studies demonstrated different outcomes in an atherosclerotic mouse model deficient for the Th2-related cytokines, the role of Th2 cells await further investigation. Furthermore, since most of these studies were performed in C57BL/6 mice that are genetically prone to Th1-, rather than Th2-mediated immunity (107), the mouse strain used might not be appropriate to produce convincing results, dependent upon the experimental conditions. Therefore, it is not surprising that no agreed role of Th2 could be derived. How Th2 responses affect the atherosclerotic progress need to be verified in other animal models.

#### Th17 Cells

Th17 cells develop in response to TGF-β with either IL-6 and IL-21, or IL-1 and IL-23. With the expression of defining transcription factors STAT3, retinoic acid receptor-related orphan receptors gamma (RORγ), and alpha (RORα), Th17 cells secrete the signature cytokines IL-17A and IL-17F along with IL-22 and IL-23 (108, 109). Both IFN-γ and IL-4, the main stimulators of Th1 and Th2 differentiation, respectively, inhibit Th17 differentiation.

The role of Th17 cells in atherosclerosis is also inconclusive, although they were found in the vascular lesions in both animal models and patients (110–112). Likewise, inconsistent data have been derived from the knockout mouse models in different studies. For example, IL-17A^−/−^Ldlr^−/−^ mice demonstrated a similar plaque burden within the aortic roots or descending aorta with that of Ldlr^−/−^ mice although their aortic macrophages, CD11b^+^DCs, and T cells were reduced (113), whereas IL-17A^−/−^ ApoE^−/−^ or IL-17RA^−/−^ApoE^−/−^ mice manifested reduced atherosclerosis by 35 or 25%, respectively, indicating a pro-atherosclerotic role of IL-17A signaling (114). Two recent studies, however, described atheroprotective roles of IL-17A, because overexpression of IL-17A in SOCS3^−/−^Ldlr^−/−^ mice resulted in reduction of lesion size by 50% within the aortic roots, accompanied by reduced vascular T cell infiltration (115). Consistently, another study showed significantly more atherosclerosis and vulnerable plaque phenotype in IL-17A^−/−^ApoE^−/−^ mice (116). Interestingly, these atheroprotective findings in animal models were corroborated in human studies where enhanced STAT3 phosphorylation and elevated IL-17 concentration were detected in patients with a better clinical phenotype, accompanied by a decrease in macrophage number and an increase in SMC deposit (115, 117).

In addition to the conflicting effects with genetic deletion mentioned above, various methods used to suppress the activity of existing IL-17A also yielded inconsistent results. For example, one study reported that the administration of rat anti-mouse-IL-17A antibody in ApoE^−/−^ mice led to a 50% reduction in aortic root lesions but increased collagen contents with decreased cellularity, suggesting better recovery (118). Consistently, blockade of IL-17A with an Fc-containing fusion protein in ApoE^−/−^ mice resulted in a reduction of lesion size by 54% throughout the aorta (110). However, another study also used rat anti-mouse-IL-17A antibodies in the experiments, where they observed reduced aortic root plaque by 43% without evidence of diminished IL-17A signaling, indicating IL-17A independent plaque reduction (119). Interestingly, when a mouse anti-mouse-IL-17A antibody were used by the same group, no improvement in atherosclerotic burden was observed, although IL-17A production was suppressed significantly (119), indicating that the exogenous antibody-induced atheroprotective effects might not be derived from suppressed levels of IL-17A but from the species in which the anti-IL-17A antibody was produced (119, 120). Therefore, the roles of Th17 cells and IL-17A in atherosclerosis is still an obscure area for intensive study.

#### Tregs

Tregs develop in response to TGF-β, express the forkhead/winged helix transcription factor (Foxp3), and secrete the signature cytokine IL-10. Having critical roles in the inhibition of inflammation and the regulation of adaptive immune responses, Tregs are potent atheroprotective cells and exert their function by multiple effector mechanisms (121).

Tregs have been detected in normal aortic tissues of mouse and human samples and their numbers were reduced in atherosclerotic mice and cardiovascular patients compared to healthy controls (122, 123). The adoptive transfer of Tregs ameliorated atherosclerosis via CD40/CD40L interaction (124, 125), whereas depletion of Tregs exacerbated the vascular inflammation with disturbed lipid phenotype (126), confirming an atheroprotective role for Tregs.

Apart from direct roles at cellular levels, the atheroprotective effects of Tregs are also manifested in their signature cytokines. IL-10 has been reported to induce potent atheroprotective activities (124, 127). Administration of IL-10 alleviated atherosclerosis in Ldlr^−/−^ mice (128), whereas inhibition of IL-10 by a genetic approach or antibody blocking aggravated the disease (129, 130), which could be caused either directly by the atheroprotective role of IL-10 or indirectly by the enhanced Th1 differentiation and macrophages accumulation in atherosclerotic lesions in the absence of IL-10, or a combination of both (127, 129, 131). In any case, the atheroprotective effects of Tregs seem to require IL-10, which was proven to have a suppressive role in the establishment of atherosclerotic lesions (127, 129).

TGF-β, another anti-inflammatory cytokine produced by Tregs in atherosclerotic plaques, suppresses pathogenic immune responses. For example, it has been demonstrated that TGF-β by itself can help to stabilize plaque via promoting collagen biosynthesis by SMC (132, 133). Further study revealed that over-expression of TGF-β limits plaque growth via reduced expression of TNF-α and the T cell attracting cytokines MIP-1α and MIP-1β in aortic tissues (134). In addition to cytokines, a recent study implicated that IDO and IDO-catalyzed tryptophan metabolism were partly responsible for the Treg-mediated immune suppression and tolerance in the vascular tissues, which can mutually regulate one another to promote vascular tolerance and limit inflammation and atherosclerosis (135), since 3-hydroxyanthranilic acid generated in the kynurenine pathway shift the differentiation of Th effector cells toward a regulatory phenotype, thus lowering plasma lipids and decreasing atherosclerosis in Ldlr^−/−^ mice (136). Overall, these data suggest that Treg, either at cellular levels through surface protein like CD40/CD40L interaction, or together with its promoting and signature cytokines, IL-10 and TGF-β, as well as downstream dioxygenase IDO, exert strong anti-inflammatory functions and suppress lesion development in atherosclerosis.

As atherosclerosis progresses to later stages, however, the number of Treg in atherosclerotic mice reduced, and an intermediate Th1-like Treg subset (Th1/Tregs) accumulated within the aorta that is dysfunctional in suppressing atherosclerosis (137). Furthermore, single-cell RNA-sequencing (scRNAseq) and real time PCR demonstrated that this newly appeared Th1/Treg subset arise from Treg, rather than from other T-effector cells, with co-expression of both Treg and Th1 lineage genes (137), suggesting atherosclerosis could drive Treg plasticity for inflammatory Th cell conversion. Along this line, a recent study revealed that Treg in ApoE^−/−^ mice fed with a western diet lose FOXP3 expression and their immunosuppressive functions were diminished with conversion of a fraction of these cells into pro-atherogenic T follicular helper (Tfh) cells (138). Consistent with the pathogenic T cell transformation in animal models, in patients with atherosclerosis, a substantial proportion of the FOXP3^+^ T cells obtained simultaneous expression of Th17 transcription factor RORgt or Th1 transcription factor T-bet (139).

### CD8^+^T Cells in Atherosclerosis

CD8^+^T cells are a subpopulation of T cells that exhibit cytotoxic activity once activated. Several lines of data indicate that CD8^+^T cells are related to atherosclerosis and vulnerable plaque development (140). Detected in both murine and human plaques, CD8^+^T cells may have a pro-atherogenic role since stimulation of these cells with a CD137 agonist accelerated lesion formation and promoted their recruitment to the lesion site (141). CD8^+^T cells promote atherosclerosis by perforin- and granzyme B- mediated cytotoxic mechanisms, as supported by their failure in this action following adoptive transfer of CD8^+^T cells deficient in perforin and granzyme B (142). Consistent with the effects of these cells in mice, correlative studies imply important roles of CD8^+^T cells in humans with coronary artery disease, because a predominance of CD8^+^T cells were observed around the shoulder regions and fibrous caps in advanced human lesions (143). Thus, specific depletion of cytotoxic T cells could theoretically be considered as an applicable approach to suppress their pro-atherogenic role in atherosclerosis development.

Indeed, CD8^+^T cell depletion by a specific antibody in Ldlr^−/−^ mice resulted in suppressed atherosclerotic plaque formation, accompanied by reduced macrophage aggregation within lesions (144). A further mechanistic study demonstrated that CD8^+^T cells could mobilize the granulocyte and monocyte progenitors in the bone marrow and spleen via IFN-g to increase circulating Ly6C monocytes, which migrated into vascular tissues and differentiated into the proatherogenic macrophages (144), indicating alternative mechanisms of these cytotoxic T cells in regulating innate immune cell responses in atherosclerosis.

Like CD4^+^T cells, CD8^+^T cells also have a regulatory subset. CD8^+^CD25^+^T cells have been reported to inhibit immune signaling and regulate experimental autoimmune disorders by directing their cytotoxic activity toward APCs (145). Similarly, this regulatory CD8^+^ subset in the spleen of apoE^−/−^ mice was found to have surface markers and functions of suppressor cells, whereas adoptive transfer of CD8^+^CD25^+^ T cells alleviated atherosclerotic lesions and suppressed CD4^+^ T cell proliferation and differentiation toward a pathogenic subset (146), indicating that CD8^+^CD25^+^T cells contributed to the suppression of the disease in experimental atherosclerosis.

### T Cell Complexity in Atherosclerotic Plaques From Mouse and Human

The recent development and rapid progress of single-cell technologies, such as scRNAseq and high dimensional cytometry by time of flight (CyTOF), revealed more complex T cell heterogeneity in atherosclerotic plaque than what were understood from traditional immunohistochemistry and conventional cytometry methods. These modern approaches now enable comprehensive mapping of the wide range of cell types and their phenotypes present in plaques with better accuracy at single cell levels after both proteomic and genomic analysis. While CyTOF is more powerful at discerning leukocyte subsets in the atherosclerotic aorta, scRNAseq provides more insights into their likely functions. Since the identification of Th1-like IFN-g^+^CCR5^+^Treg subset within the mouse atherosclerotic aorta for Treg plasticity (137) as mentioned before, leukocyte diversity (147), myeloid cell discrepancy (148), and macrophage heterogeneity (149, 150) in mouse atherosclerotic aorta were further assessed by scRNAseq and CyTOF. 97% of all aortic leucocyte populations could be defined into 21 clusters, including 10 myeloid cell clusters and 11 lymphoid clusters. Within the lymphoid clusters, a T cell subset manifested the highest complexity (7 T cells, 4 B cells, and 1NK) (147). In another study of plaque leucocyte composition biased in the use of more myeloid than lymphoid markers in the multi-panel of antibodies selected for the CyTOF, 20 clusters of myeloid cells and 13 subsets of lymphoid cells were identified (148). Out of the 13 lymphoid cell subsets, 3 subsets of CD4^+^T cells, 4 subsets of CD8^+^T cells, and 2 subsets of gd T cells were found, which likely include memory, regulatory, and effector subsets (148).

Parallel to mice, human atherosclerotic plaques were also diversified in immune cell composition, mostly populated by T cells and monocytes via CyTOF tested on leukocytes isolated from a human carotid endarterectomy specimen (147). A more recent study revealed distinct features of both T cells and macrophages in carotid artery plaques of patients and identified specific immune dysregulation associated with clinical cardiovascular events (151). Plaques from symptomatic patients were characterized by a distinct subset of CD4^+^T cells and T cells that were activated (high levels of activation of markers, i.e., CD69, CD38, CCR5, and HLA-DR) and differentiated (low levels of co-stimulatory molecules, i.e., CD28, CD27, and CD127). Moreover, some T cell subsets in plaques presented high PD-1 levels, suggesting that these subsets may simultaneously initiate an exhaustion reprogramming in response to plaque chronic inflammation (151). Additionally, macrophages from plaques contained alternatively activated phenotypes, including subsets associated with plaque vulnerability. In plaques from asymptomatic patients, T cells and macrophages were activated and displayed evidence of IL-1β signaling (151). The identification of more specific features or markers of innate and adaptive immune cell subsets in atherosclerotic plaques may enable the design of more precisely tailored cardiovascular immunotherapies.

## Markers and Tools in Atherosclerosis From Mouse and Human

Given the multiple roles of constantly identified new and old DC/T cell subsets during atherosclerotic development, multiple studies have addressed whether the number and surface molecules of these cells correlate with atherosclerosis severity and thus may serve as biomarkers or tools for the disease. In a CyTOF-based comprehensive mapping of the immune cell subsets within atherosclerotic aorta from ApoE^−/−^ mice, DCs were further analyzed in the subsets of pDC (SiglecH^+^B220^+^), cDC1 (CD11b^low^CD11c^+^MHCII^hi^CD172a^−^CD103^+^), and cDC2 (CD11b^+^CD11c^+^MHCII^hi^CD172a^+^) according to the expression of their cell makers (148). When ApoE^−/−^ mice were fed a high fat diet, cDC1 remained unchanged whereas cDC2 arterial contents were slightly decreased and pDCs were significantly increased (148), demonstrating the diagnostic value of a pDC subset. Using a scRNAseq approach, another DC cluster (*Cd209a*^+^*Cd74*^+^*Flt3*^+^*H2-Eb1*^+^DC cluster) containing both Cd209a^+^monocyte-derived DCs and putative mature/classical DCs, together with two macrophage populations (inflammatory macrophages enriched in *IL1b* and TREM2^high^ macrophages), were detectable in mouse atherosclerotic aorta, where DCs as a major atherosclerosis-associated cell population represented 14.9% of total CD45^+^ cells and showed strong expression of *IL1b* (149). Other specific markers enriched in the DC cluster but not in macrophages included *Flt3, Ifi30* (g-interferon-inducible lysosomal thiol reductase), *Napsa* (Napsin A), *Itgb7* (intergrin b7), *Syngr2* (synaptogyrin-2), *Clec10a*, and *Ahr* (aryl hydrocarbon receptor) (149).

In humans, pDCs express the surface maker blood dendritic cell antigen (BDCA)-2, while myeloid DCs (mDCs) could be categorized into mDC1 subsets with BDCA-1 and mDC2 subsets with BDCA-3 (152). Human mDC2 share most features with murine CD8^+^DCs, while mDC1 represent the human counterpart of murine CD8^−^DC (153). Absolute and relative numbers of circulating pDCs were dramatically lower in patients with coronary artery disease than in controls. The decrease tended to be more pronounced in unstable coronary syndromes and extensive coronary artery disease, suggesting a possible role of pDCs as a prognostic tool in plaque progression and rupture (154). The percentage and absolute number of mDC2 were decreased in patients with atherosclerotic complications when compared with healthy controls, which implied that decreased circulating mDC2 could possibly serve as a marker of disease severity (155). Additionally, the absolute number of Lin1^−^HLA-DR^+^ peripheral-blood DCs and Lin1^−^HLA-DR^+^CD11c^+^mDC, but not pDCs, were increased in patients with coronary artery disease (156). In contrast, circulating mDCs, but not pDC precursors, were decreased in patients with unstable coronary artery disease via immunohistochemical (157) or FACS (158) examination. Other studies showed that a significant decrease of circulating DC precursors, like myeloid DC precursors, plasmacytoid DC precursors, and total DC precursors, were identified as an independent predictor of the presence of, and subsequent therapeutic procedure in, stable coronary artery disease (159, 160).

Interestingly, some studies observed that decreased circulating DC precursors in coronary artery disease were accompanied by a resident DC increase in the inflamed plaques and infarct myocardium, inferring that the recruitment of circulating DC precursors to inflamed plaques was a part of the underlying disease process (157, 160). In addition, detection of activated triggering receptors expressed on myeloid cells (TREM)-1^+^DCs and elevated proportions of CD303^+^pDCs in plaque indicate the vulnerability of atherosclerosis in humans (161). With a finer delineation of circulating DC subsets and precursors, their abundance, as well as activated receptor, may prove to be useful as potential biomarkers of atherosclerosis.

In addition to DCs, other cell types, such as inflammatory T cells and master cells in atherosclerotic lesions, also correlate with disease severity, indicating a complex interplay of different cell types for plaque destabilization. Therefore, modulating the interaction of local T cells with other immune cells and preventing their invasion into the plaque might be a therapeutic tool for plaque stabilization (162). Consistently, accumulating evidence indicates that on evaluation of the feasibility of DCs and T cells as a diagnostic tool, the subsets of blood CD4^+^, CD8^+^, and CD4^+^CD25^+^Foxp3^+^T cells and the ratio of CD4 to CD8 cells hold promise as biomarkers of coronary artery disease (163).

## Potential Therapeutic Applications for Atherosclerosis

Given the presence of both protective and pathogenic immune responses in the vascular tissues involving DCs and, inevitably, their adaptive partners, T cells, therapeutic strategies designed can be those attempting to either boost protective pathways or inhibit pathogenic pathways implemented by these two types of cells.

### DC Therapeutics for Atherosclerosis

To harness DCs as a therapeutic vaccine, GM-DCs (bone marrow-derived DCs cultured with GM-CSF) with an immature phenotype were loaded with oxLDL or ApoB (164, 165), potential autoantigen in atherosclerosis, and adoptively transferred to Ldlr^−/−^ mice. These treatments led to a significant suppression in atherosclerotic lesion development and diminished oxLDL/ApoB-specific T cells, but enhanced oxLDL/ApoB -specific IgG levels, as well as Treg induction, indicating an antigen specific immunotolerant role for immature DCs in atherogenesis. To take advantage of the tolerogenic natures of immature DCs, a recent study utilized IL-37 to inhibit the maturation of DCs (166). In contrast to immature DCs, ApoE^−/−^ mice receiving mature GM-DCs loaded with the modified autoantigen, MDA-LDL (malondialdehyde-modified LDL), suffered from aggravated atherosclerosis with a 40% increase in lesion size, augmented vascular cell adhesion molecule 1 (VCAM-1) expression, and increased MDA-LDL-specific IgG/IgM levels, but no induced Tregs (167). Since transfer of MDA-LDL-reactive T cells has also been demonstrated to accelerate atherosclerosis (168), these results indicated that monocyte-derived GM-DCs are atherogenic in their mature state, but atheroprotective when they are immature. Likewise, DCs loaded with another auto-peptide, β-galactosidase (β-gal), could give rise to a similar result. For example, in a separated study, increased lesion development was observed when ApoE^−/−^ mice expressing β-gal in arterial specific tissues received GM-DCs loaded with β-gal-specific peptides (169).

Despite the success found in many cases, the above DC immunization approaches have several limitations, leaving plenty of room for improvement. Firstly, the BM-DCs cultured *in vitro* constitute a heterozygous population of DCs and macrophages, whose immune stimulatory capacity is far less efficient than that of *bona fide* DCs (170). Even purification of DCs using conventional methods such as CD11c^+^ microbeads cannot obtain a uniform DC population as macrophages in the cultures also express CD11c (170). Therefore, alternative methods with a purer DC yield should be sought. Secondly, the maturation status of DCs was not well-evaluated before immunization. It is well-established that immature DCs promote T-cell tolerance via deletion of self-reactive T cells and induction of Tregs (171), whereas mature DCs induce Th1 polarization for immune activation (172). It would be interesting to evaluate the effects of adoptively transferred purer DCs pulsed with modified LDL (e.g., oxLDL, MDA-LDL, β-gal, etc.) after exposure to inhibitive cytokines such as IL-10, TGF-b, or IL-37 etc. in atherosclerotic animal models.

Given all the shortcomings that come with the cultured DCs, better immunogenic cellular sources *in vivo* and atherogenic autoantigen are needed. Fortunately, the pioneering works by Steinman and Nussenzweig could shed an interesting light in this direction, where antigens were directly linked to antibodies against endocytic surface receptors that are highly expressed in particular DC subsets *in vivo* (173). For example, targeting a self-antigen fused to an antibody against CD8^+^ DC abundant receptor CD205/DEC205 resulted in tolerance induction, whereas strong immunity could be elicited if the same DC subset was recognized by an antibody linked to the foreign antigen in the presence of an adjuvant TLR agonist, such as PolyI:C (172). Furthermore, many other DC subset-specific antibodies, such as anti-CD207, anti-Clec9A, and anti-PDCA1/BST2/CD317, have also been identified for targeting proteins/peptides to specific DC subsets (174–178). It follows that once appropriate protein/peptide targets for atherosclerosis or specific DC subsets responsible for inflammation control in vascular tissues are identified, the DC-targeting-antibody approach will be tested in animal models for its potency in the prevention of atherosclerosis.

Along this line, different atherosclerosis-related autoantigens and DCs with various immunogenicity were tested to develop effective DC-based vaccination strategies against this vascular inflammatory disease. For example, adoptive transfer of the tolerogenic ApoB100 pulsed DCs that had been pre-exposed to immunosuppressive cytokine IL-10 led to decreased lesion progression, alleviated systemic inflammation, and reduced CD4^+^T cells infiltration in human ApoB100 transgenic Ldlr^−/−^ mice (165). Moreover, to effectively target the particular atherosclerosis-related DC subset *in vivo* and the pathway involved, a DEC205-DC targeted DNA vaccine against th eCX3CR1 chemokine pathway was administrated in ApoE^−/−^ mice. Strong cDC1 subset-mediated immune responses and attenuated atherosclerotic lesions were observed in the brachiocephalic artery with reduced macrophage infiltration, but there was no effect on SMC migration in the mice, indicating a potential therapeutic option for more effective treatment of atherosclerosis (179). Collectively, these studies suggest that DC-based vaccination could well be a more powerful tactic in the treatment of atherosclerosis if the right cells and/or molecules are targeted. To this end, new approaches are needed to find the novel atherogenic self-antigens or defective immunological pathways in atherosclerotic lesions to design efficient immunosuppressive vaccines.

In addition to specific antigenic signal 1 mentioned above, interference with signal 2 or immunological check point molecules between the DC/T cell cross-talk is also an effectively targeted immunosuppressive strategy for the attenuation of vascular inflammation. For example, combined deletion of CD80 and CD86 in Ldlr^−/−^mice was used to result in an alleviated atherosclerotic burden (180). Since cytotoxic T-lymphocyte-associated protein 4 (CTLA4) competes with CD28 for binding to CD80/86, biological therapies based on CTLA4 are approved for rheumatoid arthritis and kidney transplantation. This inhibitive pathway was also exploited for the treatment of atherosclerosis in mice. For example, translational studies using CTLA-Ig fusion protein Abatacept, which mimics CTLA4 function, have shown promising outcomes, as researchers have observed decreased intimal hyperplasia and inflammation with this CTLA4 analog (181). Along the same line, chronic tissue inflammation in the atherosclerotic plaque was found to be associated with an overreactive programmed cell death protein 1 (PD-1) checkpoint, and vessel-wall-embedded DCs from patients with giant cell arteritis, another type of vascular inflammation, were PD-L1^lo^ (182). Thus, immunoinhibitory signals that affect multiple stages of vascular inflammation can be utilized in DC therapy to regulate local immune responses in the aortic tissues.

### T Cell Therapeutics for Atherosclerosis

The most effective DC therapies outlined above are ultimately mediated via T cells. Given the pivotal roles of Tregs in keeping immunological balance *in vivo*, a great deal of research efforts is now focused on developing Treg-based therapies to adjust the dysregulated immune responses in atherosclerosis.

A possible strategy to use Tregs for therapy is the adoptive transfer of *ex vivo* expanded Tregs. Experiments with animals demonstrated a remarkable reduction in atherosclerosis in response to Treg injection *in vivo*, suggesting that a similar effect could be obtained in patients (124). This procedure, however, requires large numbers of Tregs, which is still achievable from the isolation of peripheral blood for Foxp3^+^CD4^+^T cells with subsequent expansion *ex vivo* for therapy using appropriate cytokine mixtures (183, 184). Treg based therapy turned out to be effective with no significant adverse effects reported so far. Unfortunately, in human trials, due to the influence of multiple factors, there has not been enough data so far to evaluate the long-term effects that transferred Tregs will bring. Therefore, more studies from human trials are required to design effective therapies for atherosclerotic disease.

In addition to the adoptive transfer of *ex vivo* expanded Tregs in general, multiple studies in animal models demonstrated that the induction of polyclonal or specific Tregs against autoantigens such as oxLDL, ApoB100, and HSP60 *in vivo* suppressed atherosclerosis development and/or progression (185). Indeed, the activation of antigen-specific Tregs in the disease-bearing host could be very effective in suppressing atherosclerosis. For example, a study showed that oral or nasal administration of HSP60 inhibits atherosclerosis formation, just as the adoptive transfer of HSP60-specific CD4^+^CD25^high^ cells induced through HSP60-loaded DCs *in vitro* does, indicating the antigen specific Tregs could be induced by DCs *in vivo* (186). One of the most promising atherosclerosis specific antigens is ApoB100, and some studies have shown that ApoB100 peptide-based vaccines induced Treg to inhibit atherosclerosis in mice (165, 187). Therefore, it is not surprising to see that vaccination protocols using ApoB100 are being developed in human clinical trials (188). Although many other candidates for atherosclerosis-related antigens, like oxLDL and HSP60, have also been studied, so far the dominant relevant antigens that could effectively induce Tregs have still not been uncovered.

Following their induction by appropriate antigens, various attempts have been made to improve the survival and efficacy of endogenous Treg *in vivo* (189). Low dose IL-2 or IL-2 in combination with an anti-IL-2 antibody was found to enhance the stability of Treg, and preferentially promote their expansion (190). This therapeutic strategy hopefully can reduce many T cell-mediated pathologies in vascular tissues or where an increased Treg response can ease inflammatory injuries. In addition to cytokine IL-2, an immunosuppressive drug has also been put forward to sustain Tregs in atherosclerotic plaques. For example, the clinical administration of mycophenolate mofetil in small groups of patients with atherosclerotic carotid artery stenosis was found to result in enhanced Treg differentiation, but a reduced effector T cell activation in carotid atherosclerotic lesions (191).

Despite their profound outcomes in animal studies, the safety and potency of Treg-based therapies in human atherosclerosis needs to be carefully investigated, and many critical problems remain to be solved. A major obstacle in Treg-based immunotherapy is the stability of Tregs during the longer culture period *in vitro* for large expansion, and the following important scientific questions remain: Would the Tregs still keep their immune suppressive features or could they lose Foxp3 expression after experiencing such extensive proliferative cycling (192)? In addition, would Treg transfer alone be enough to suppress the chronic inflammation in the diseased site? If not, comprehensive strategies with combined pro-inflammatory T cell deletion might be necessary.

## Conclusions and Future Directions

Atherosclerosis involves complex activity characterized by the activation of endothelial cells and monocytes/macrophages followed by the transmigration of antigen-specific DCs and T cells into the intima. A recent study suggested that local “trained immunity” and immunometabolism of these two cell types have emerged as new actors during atherogenesis (193). Therefore, restoration of their defective functions or understanding their distinct immunological features could lead to effective treatment of patients with cardiovascular diseases. Unfortunately, given the high DC plasticity, many so called DC-specific mouse models lack specificity, which makes the contribution of these leucocytes to atherosclerosis difficult to pinpoint. Recent new studies, however, on DC subset specific transcription factors such as IRF4 (194) and Zbtb46 (195, 196), or with new technologies such as intravital imaging (197), scRNAseq, and CyTOF (148, 149), have generated some interesting outcomes. With improved knowledge regarding the specific roles of DC/T cells in vascular inflammation, therapeutic strategies in development encompass the vaccine-based therapies, adoptive transfer therapies, and antibody-based immune targeting therapies that aim for specific co-stimulation molecules or subset markers (Table 3). More importantly, these therapies on atherosclerosis also start to yield important consequences for other autoimmune patients.

**Table 3:** Therapeutics strategies for atherosclerosis.

**Therapies**	**Methods**	**Objectives**	**References**
**DCs**			
Immature DCs	Injecting purified immature DC cultured *in vitro*	To promote T cell tolerance and induce Tregs	(166, 170)
Self-Ag pulsed immature DCs	Injecting cultured immature DCs loaded with AS-related Ag *in vitro*	To induce AS-specific tolerance	(164, 165)
DC specific Abs conjugated with AS-related self Ag	Injecting AS-related Ag conjugated to Ab against specific markers on DC subsets *in vivo*, such as CD205/DEC205, CD207, Clec9A and PDCA1/BST2/CD317 etc.	To target specific AS-related DC subset *in vivo* with self Ag for tolerance induction	(175–178)
**T cells**			
Th17 antibody	Administration of Anti-IL-17 Ab	To block Th17 responses	(118)
Treg inducing self-peptides	Administration of peptides of oxLDL or ApoB 100 via nasal or oral application	To induce Treg differentiation	(187, 188)
T cell conditioning reagents	Injecting low-dose IL-2 or T cell suppressive drugs such as mycophenolate mofetil	To improve Treg activity *in vivo*	(190, 191)
T cell costimulatory molecule-targeting strategies	Translational administration of CTLA4-Ig fusion protein, Abatacept	To mimics CTLA4 function and compete with CD28 for binding to CD80/CD86 for immune suppression	(181)
	Transfer of vessel-wall-embedded PD-L1^lo^ DCs from patients with giant cell arteritis	To reduce PD1/PDL1 co-inhibitory pathway for immune enhancement	(182)

*Treg, regulatory T cells; Ag, antigen; AS, atherosclerosis; Ab, antibody; Ig, immunoglobin*.

Although considerable progress has been made in our understanding of the behavior and functions of arterial DC and T cell subsets, there is still a great deal to learn and improve on the road ahead for therapeutic gains. Firstly, as antigen presentation can occur within the arterial wall, the precise contributions of local VALT vs. systemic SLO antigen presentation to the etiology of atherosclerosis are unclear. Therefore, comparative studies of the immunological features between VALT and SLO during atherogenesis may provide valuable information underlying the pathophysiology of atherosclerosis, which might lead to better treatment strategies. Secondly, the population of DCs in atherosclerotic plaques shows significant heterogeneity with different or sometimes opposing functionality, and different subsets of DCs are widely uncharacterized in affected humans. Identification of specific intraplaque arterial DC subsets and their antigen presentation capacity with high throughput omics at single cell levels may help to deliver vaccines to the right cells via monoclonal antibody for more effective control of local inflammation *in vivo*. Moreover, in terms of the effector cells downstream of these vascular DCs, the antigenic reactivity of the T cells in atherosclerotic lesions remains to be well-characterized. Although tetramers containing the newly identified MHCII-restricted epitopes in ApoB have recently been used to detect antigen-specific T cells in atherosclerosis development (139), safe translation of these approaches to the clinical setting awaits thorough investigation. Only then can a better immunosuppressive effect be achieved to open up new therapeutic avenues for clinical applications.

## Author Contributions

LS wrote the initial draft of the manuscript. WZ and YZ made the figures. YX supervised the writing, analyzed, and refined the manuscript. All authors listed have made a substantial, direct, and intellectual contribution to the work, and approved it for publication.

## Conflict of Interest

The authors declare that the research was conducted in the absence of any commercial or financial relationships that could be construed as a potential conflict of interest.
